# Supplementary material for: Titanium Dioxide Thin Films Obtained by Atomic Layer Deposition Promotes Osteoblasts’ Viability and Differentiation Potential While Inhibiting Osteoclast Activity—Potential Application for Osteoporotic Bone Regeneration
Source: Materials (Basel). 2020 Oct 28;13(21):4817. doi: 10.3390/ma13214817 (PMC7662580; doi:10.3390/ma13214817)
Supplement: Supplementary file 1 [file materials-13-04817-s001.pdf]

materials

MDPI

Supplementary Materials:

Titanium Dioxide Thin Films Obtained by Atomic Layer Deposition Promotes Osteoblasts' Viability and Differentiation Potential While Inhibiting Osteoclast Activity—Potential Application for Osteoporotic Bone Regeneration

Table s1. The list of oligonucleotides used for determination of specific transcripts.

Gene Name	Gene Abbreviation	Primer	Sequence 5'-3'	Amplicon ength [bp]	Annealing Temp. [^{0}C]	Accesion no.
Osteopontin	*Opn*	F:<br>R:	AGACCATGCAGAGAGCGAG<br>GCCCTTTCCGTTGTTGTCCT	340	57,3	NM_001204203.1
Osteocalcin	*Ocl*	F:<br>R:	GGTGCAGACCTAGCAGACACA<br>CGCTGGGCTTGGCATCTGTAA	100	57	NM_001032298.3
Collagen type I	*Coll1*	F:<br>R:	CAGGGTATTGCTGGACAACGTG<br>GGACCTTGTTTGCCAGGTTCA	107	61,4	NM_007742.4
Runt related transcription factor 2	*Runx-2*	F:<br>R:	TCCGAAATGCCTCTGCTGTT<br>GCCACTTGGGGAGGATTTGT	130	58,8	NM_001271630.1
Glyceraldehyde-3-phosphate dehydrogenase	*Gapdh*	F:<br>R:	TGCACCACCAACTGCTTAG<br>GGATGCAGGGATGATGTTC	177	60	XM_017321385.1
miR-7a-5p	*miR-7*	F:	TGGAAGACTAGTGATTTTGTTGT	*	58,8	MIMAT0000677
miR-17	*miR-17*	F:	CAAAGTGCTTACAGTGCAGGTAG	*	58,8	MIMAT0013084
miR-21-5p	*miR-21*	F:	TAGCTTATCAGACTGATGTTGA	*	58,8	MIMAT0000076
miR-124-3p	*miR-124*	F:	TAAGGCACGCGGTGAATGCCAA	*	58,8	MIMAT0000422

* Due to poly (A) sequence it is not possible to predict the actual product size

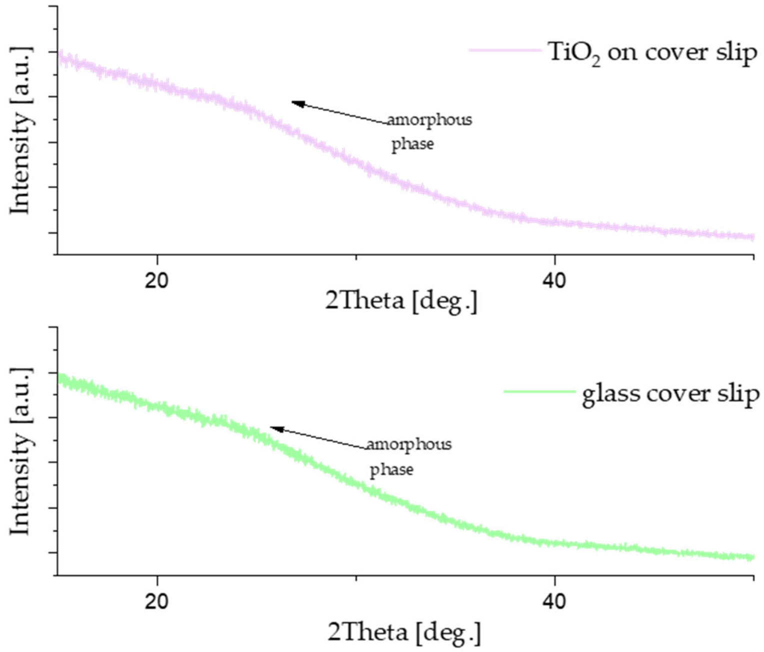

Figure s1. The XRD data of: TiO_2 coating obtained by ALD on the cover slip (top) and blank cover slip as a reference (bottom).
